# Ni stabilized rock-salt structured CoO; Co_1−*x*_Ni_*x*_O: tuning of e_g_ electrons to develop a novel OER catalyst†

**DOI:** 10.1039/d0ra03050c

**Published:** 2020-05-07

**Authors:** Rakesh Mondal, Himanshu Ratnawat, Sarvesh Kumar, Anil Kumar, Preetam Singh

**Affiliations:** Department of Ceramic Engineering, Indian Institute of Technology (Banaras Hindu University) Varanasi Uttar Pradesh India-221005 preetamsingh.cer@itbhu.ac.in preetamchem@gmail.com +91-9473720659

## Abstract

The oxygen evolution reaction (OER) is a key half-reaction in hydrogen–oxygen electrolysers that is very important for efficient electrochemical energy generation, storage and fuel production that offers a clean alternative to fissile fuel combustion based energy systems. Several transition metal containing perovskites were recently explored for the development of superior OER catalysts, and their activity was correlated with the applied potentials at a specific current density to e_g_ electron density present in the materials. The rock salt structure is envisaged here as a model host structure similar to perovskite to tune the e_g_ electrons to obtain superior electro-catalytic activity. Incorporation of Ni into CoO lattices helps to stabilize the rock salt structure and modulate the e_g_ electrons to develop superior OER and ORR electrocatalysts. Nickel doped rock salt structured CoO, Ni_*x*_Co_1−*x*_O (0 ≤ *x* ≤ 0.5), were synthesized by employing a solid state metathesis synthesis route. The compounds were characterised by powder X-ray diffraction (XRD), TGA, FT-IR and X-ray Photoelectron Spectroscopy (XPS). Ni_0.3_Co_0.7_O with 1.3 e_g_ electrons showed superior electrocatalytic activity for the oxygen evolution reaction. The overpotential for the Ni_0.3_Co_0.7_O sample was also found to be ∼0.450 V for 1 M and about ∼0.389 V at 5 M concentration of the KOH electrolyte.

## Abstract 2

Incorporation of Ni into CoO lattices helps to stabilize the rock salt structure and modulate the e_g_ electrons to develop superior OER and ORR electrocatalysts.

## Graphical abstract

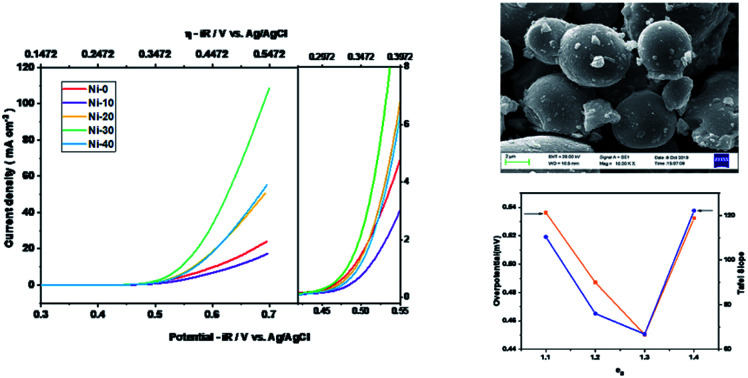

## Introduction

Efficient electrochemical energy generation, storage and fuel production offers a clean alternative to fissile fuel combustion based energy systems.^1–3^ The oxygen evolution reaction (OER) is a key half-reaction in hydrogen–oxygen electrolysers, rechargeable metal–air batteries, and regenerative fuel cells such as solid oxide electrolyser cells (SOECs). However, the OER suffers due to sluggish kinetics and the issue of overpotentials. To overcome the overpotential and sluggish kinetics of the OER, efficient catalysts are required.^4–6^ RuO_2_ and IrO_2_ are commonly considered as benchmark electrocatalysts for the OER owing to their high electrocatalytic activities toward the OER in both acidic and alkaline solutions.^7,8^ However, their low abundance and high cost inhibit their practical usage. IrO_2_ is the best electrocatalyst so far for OER. But, it is difficult to achieve dual activity; acceptable limit of simultaneous OER and oxygen reduction reaction (ORR) activities. Therefore in recent years, the research drive is motivated toward development of dual catalyst without usage of noble/costly metals. The burgeoning efforts taken by materials chemist to develop inexpensive materials with high electrocatalytic activity and stability for OER or preferably for both OER and ORR, it is still remain as a daunting scientific challenge. However, recent studies show that the electrolysis of water is generally preferred in alkaline medium over acidic medium due to higher stability of oxide materials towards corrosion.^9–11^

Transition metal oxides such as Co_3_O_4_, MnO_2_, NiCo_2_O_4,_ LaNiO_3_, LaCoO_3_, SrCoO_3−*δ*_ in the form of spinel and perovskite structure are reported to exhibit relatively good OER catalytic activity.^12,13^ Several transition metal containing perovskite were recently explored for development of superior OER catalyst, and their activity were correlated with the applied over-potentials at a specific current density to e_g_ electrons density present in the materials.^14^ Ba_0.5_Sr_0.5_Co_0.8_Fe_0.2_O_3−*δ*_ (BSCF) was reported as the best OER catalyst with e_g_ electron (e_g_ = ∼1.2) at the peak of the volcanic graph.^14^ The systematic investigation of Co_3_O_4_ based spinels, demonstrated the individual roles of Co^2+^ ions and Co^3+^ ions during OER and it was observed that Co^2+^ and Co^3+^ ions were differently responsible for OER activity in the Co_3_O_4_ system and confirmed that the divalent Co^2+^ dominated the OER activity.^15^

Perovskite and spinel-type structures accommodate transition metal ions in different oxidation states making it difficult to get a uniform Co^2+^ oxidation state across the structure with ∼1.2 e_g_ electron. Here we envisaged Ni stabilized rock salt structured CoO in the form of Ni_*x*_Co_1−*x*_O as a model host structure to tune the e_g_ electron concentration keeping entire Co in 2+ oxidation state. Here in this manuscript, we present the stabilization of CoO in rock salt structure by doping of Ni and study of their electrocatalytic OER activity in alkaline media.

## Experimental

### Materials synthesis and characterizations

Solid state ceramic synthesis route was used to synthesize crystalline Ni doped cobalt oxides as the solid state route in general results more thermodynamically stable compound. Ni(OH)_2_·NiCO_3_·4H_2_O and CoCO_3_ were taken as suitable precursor because metathesis (simultaneous decomposition) of carbonates can result solid solutions of cobalt and nickel oxides. The precursors are taken in stoichiometric ratio and mixed in agate mortar–pestle arrangement for about 40 minutes. The mixture is then fired at a temperature of 1050 °C for 12 hours. The sample was heated twice to get single phase materials. The samples were denoted as Ni-10 to Ni-50 for 10 to 50% Ni doped cobalt oxide. The undoped cobalt oxide was denoted as Ni-0. The phase formation was studied through Rigaku Miniflex desktop X-ray Diffractometer (XRD) with Cu-Kα radiation (*λ* = 1.54 Å) in the range 2*θ* ∼ 20–90° with a step size of 0.02°. The structures were refined by Rietveld refinement method using FULLPROF suite software and cubic CoO rock salt structure (space group: *Fm*3̄*m*, no. 225) was taken as model structure. The microstructures of the sintered samples were investigated by using scanning electron microscope (EVO – Scanning Electron Microscope MA15/18). The average grain size was calculated using the linear intercept method. The composition of the compounds was examined by energy dispersive X-ray (EDX) spectroscopy with a probe attached to the scanning electron microscope. Infrared spectra of the samples were recorded using Nicolet iS5 FTIR spectrometer in the range of 400 to 4000 cm^−1^. X-ray photo-electron spectroscopy (XPS) studies were carried out to investigate the electronics structure of the materials. XPS of the sample were carried out by Thermo Scientific Multilab 2000 instrument using Al Kα radiation operated at 150 W. Binding energies reported here are with reference to C (1s) at 284.5 eV and they are accurate within 0.1 eV.

### Electrochemical studies

The electrochemical measurements were carried out using Nova 2.0 Autolab. The catalyst ink was prepared by homogenizing 12 mg of catalyst, 6 mg of carbon material and 100 μL of Nafion® ionomer solution (0.26 mg mL^−1^) in 3 mL of water under an ultrasonication bath for 40 min. To investigate the activity of the electrocatalyst, an aliquot of 10 μL of homogenized catalyst ink was deposited by a micro pipette onto the surface of a glassy carbon (GC) electrode with a geometric area of 7.06 mm^2^, the electrode was polished to a mirror-like appearance and dried under an IR lamp. The catalyst load was typically 566 μg cm^−2^ for the GC.

Linear sweep voltammetry (LSV), cyclic voltammetry (CV) and electrochemical impedance spectroscopy (EIS) in a conventional three-electrode arrangement were used to determine the electrochemical characteristics of the prepared electrocatalysts were measured by Metrohm Autolab (PGSTAT204) equipped with FRA32M module. Electrochemical measurements were analysed using NOVA software.

Pt was used as a counter-electrode and Ag/AgCl in 3 M KCl was utilized as a reference electrode. All electrode potential values mentioned in this manuscript refer to this Ag/AgCl in 3 M KCl electrode. Potential conversion for overpotential measurements were done using the following equation:
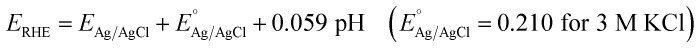
Overpotential = *E*_RHE_ − 1.23 − *iR**R* is calculated using EIS measurements. Argon saturated 1–5 M KOH solution was used as the electrolyte. The electrolyte solutions were freshly prepared prior to each set of experiments from analytical grade KOH (Lachner, Czech Republic) and deionised water.

## Results and discussions

**Fig. 1:**
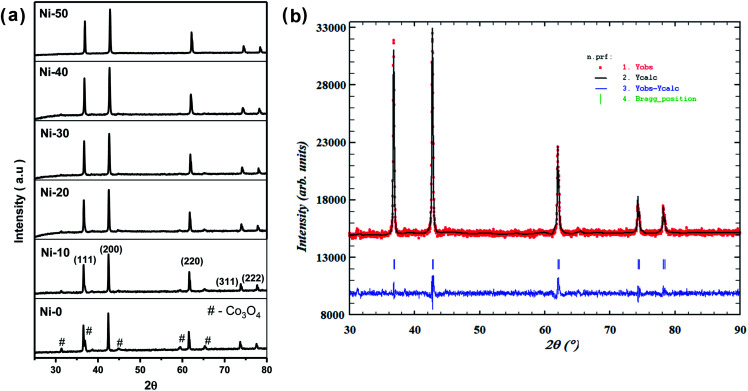
(a) X-ray diffraction patterns of Ni_*x*_Co_1−*x*_O (*x* = 0, 0.1, 0.2, 0.3, 0.4, 0.5) denoted as Ni-0 to Ni-50. (b) Rietveld refinement of XRD pattern of Ni_0.3_Co_0.7_O.

**Fig. 2:**
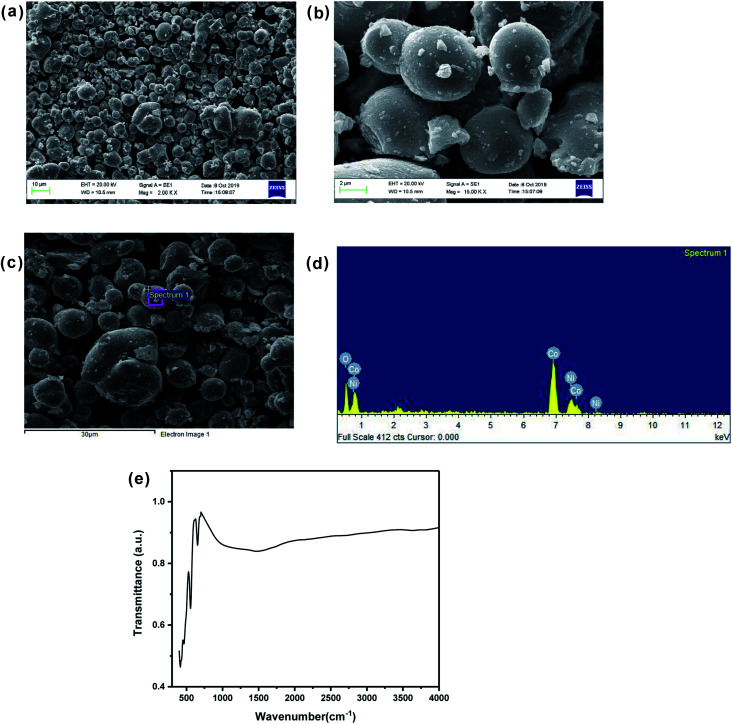
(a) Scanning electron micrograph of Ni_0.3_Co_0.7_O. (b) Scanning electron micrograph (enlarged view) of Ni_0.3_Co_0.7_O. (c) Region selected for EDX spectra. (d) EDX analysis showing stoichiometric distribution of Co, Ni and O elements. (e) FTIR spectrum of Ni_0.3_Co_0.7_O.

Fig. 1(a) shows the XRD pattern of up to 50% Ni doped cobalt oxides. Except for pure and 10% Ni doped cobalt oxide (Ni-0 and Ni-10), peaks were identified for rock salt CoO structure and for Ni-0 and Ni-10, small impurity peaks were identified for spinel Co_3_O_4_ structure in the XRD pattern. With >10% nickel doping, materials were synthesized in single phase and all the diffraction peaks were identified only to rock salt structure. Therefore, it is clear form XRD studies that more that 10% nickel substitution in cobalt oxide stabilizes the rock salt as more thermodynamically stable structure. There is continuous peak shift of (200) diffraction peaks with increasing nickel content in cobalt oxide. Thus with increasing nickel concentration, the peak shift of (200) diffraction peak toward higher 2*θ* clearly indicates substitutional incorporation of Ni ions into the CoO rock salt structure. The Rietveld refined XRD profile of Ni-30 is shown in Fig. 1(b). The observed intensities matched very well with the calculated intensities. The structural parameter derived for the refinement is given in Table 1. The lattice parameter *a* (Å) is found to vary linearly with the Ni content satisfying Vegard's law as shown in the Fig. S1 (ESI).† SEM images in Fig. 2(a) and (b) clearly shows the particle distribution and morphology of bulk 30% Ni doped cobalt oxide (Ni-30) microcrystals. The microcrystals are spherical in shape and of 2–5 micrometer in sizes. Fig. 2(c) shows the area of the sample on which EDX mapping was carried out to get the composition of the material. Fig. 2(d) shows the EDX mapping image and the data confirms that the composition of the materials (Ni : Co = 0.29 ± 0.01 : 0.7 ± 0.01)is almost same to the nominal composition utilized for the synthesis.

**Table 1:** Structural parameters of Ni_*x*_Co_1−*x*_O (*x* = 0.1 to 0.4)

Compound	Lattice parameter	*χ* ^2^	*R*_f_	*R*_Bragg_	*R*_wp_
	*a* = *b* = *c*				
Ni_0.1_Co_0.9_O	4.247946	1.219	0.518	0.896	20.6
Ni_0.2_Co_0.8_O	4.239565	0.988	0.377	0.532	15.1
Ni_0.3_Co_0.7_O	4.230142	1.15	1.57	1.87	20.7
Ni_0.4_Co_0.6_O	4.224223	3.21	0.499	0.835	20.9

The FTIR spectrum of the Ni-30 samples is shown in the Fig. 2(e). The absence of peak at 3450 cm^−1^ (assigned to hydrogen bonded hydroxyl stretching vibrations)^16^ and the peak at 1630 cm^−1^ (attributable to H–O–H bending vibration mode)^16^ shows the absence of free water in the sample. The high intensity peaks observed at 651 cm^−1^ and in the region of 407–558 cm^−1^ corresponds to Ni–O and Co–O stretching and the Ni–OH and Co–OH bending respectively.^17,18^ In general, the presence of Ni–OH bonding is known to enhance the OER activity of the sample.^19^

**Fig. 3:**
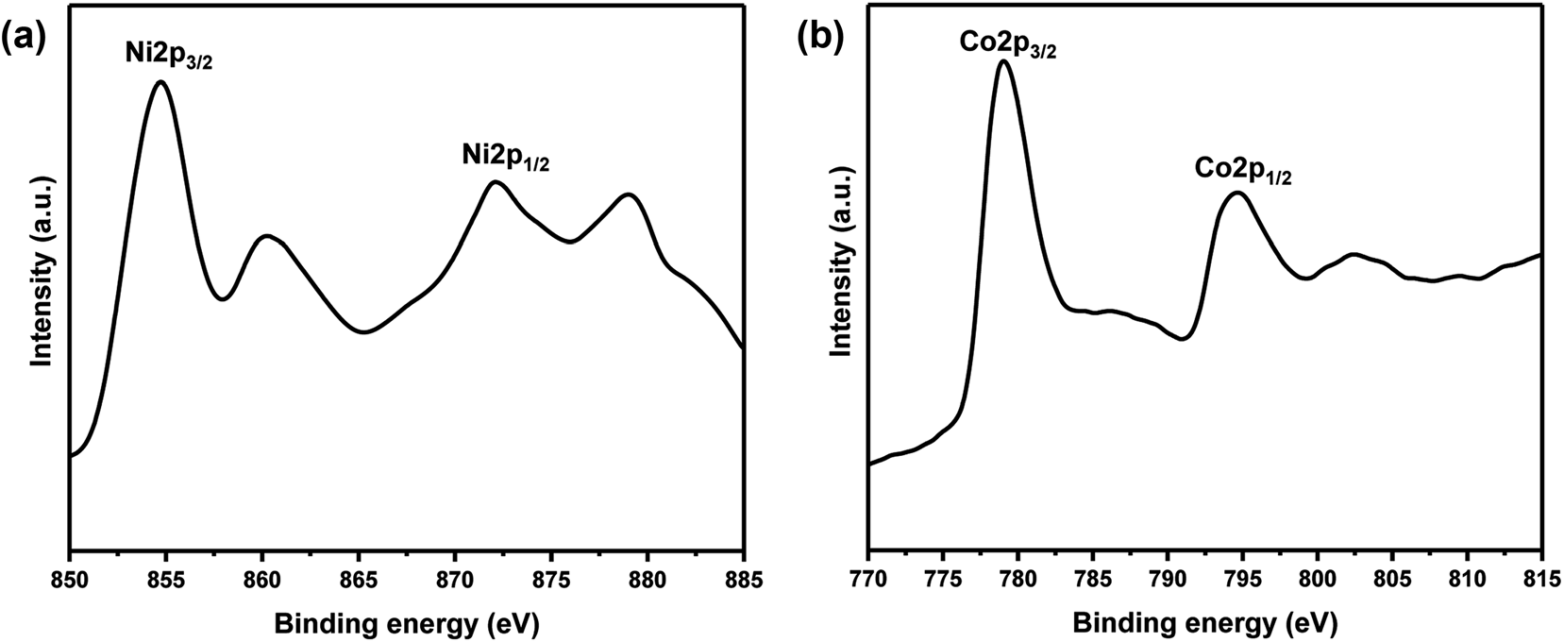
(a) Core level XPS spectrum of Ni (2p) for the sample Ni_0.3_Co_0.7_O. (b) Core level XPS spectrum of Co (2p) for the sample Ni_0.3_Co_0.7_O.

XPS measurements of core levels Ni (2p) and Co (2p) spectra of Ni-30 sample is shown in Fig. 3. The binding energy of 854.7 and 872.1 eV were observed for Ni 2p_3/2_ and Ni 2p_1/2_ peaks respectively in Fig. 3(a). The binding energies at 779.1 and 794.6 eV were observed for Co 2p_3/2_ and Co 2p_1/2_ peaks respectively in Fig. 3(b). These signal positions are typical for NiO and CoO and indicate the presence of Ni and Co atoms in the +2 oxidation state in Ni-30 sample.

Relative surface concentration is calculated from the formula:^32^Relative concentration *C*_M_ = (*I*_M_/*λ*_M_*σ*_M_*D*_M_)/∑(*I*_M_/*λ*_M_*σ*_M_*D*_M_)where *I*_M_ is the integrated intensity of the core levels (M = Ni (2p) and Co (2p)), *λ*_M_ is the mean escape depth of the respective photoelectrons, *σ*_M_ is the photoionization cross section, and *D*_M_ is the geometric factor. The photoionization cross-section values were taken from Scofield's data^33^ and mean escape depths were taken from Penn's data.^34^ The geometric factor was taken as 1, because the maximum intensity in this spectrometer is obtained at 90. Surface concentrations of Ni and Co are found in the ratio of 0.30 : 0.70 in Ni_0.3_Co_0.7_O. Thus surface compositions of Ni_0.3_Co_0.7_O almost same as the bulk composition.

### Electrochemical studies

**Fig. 4:**
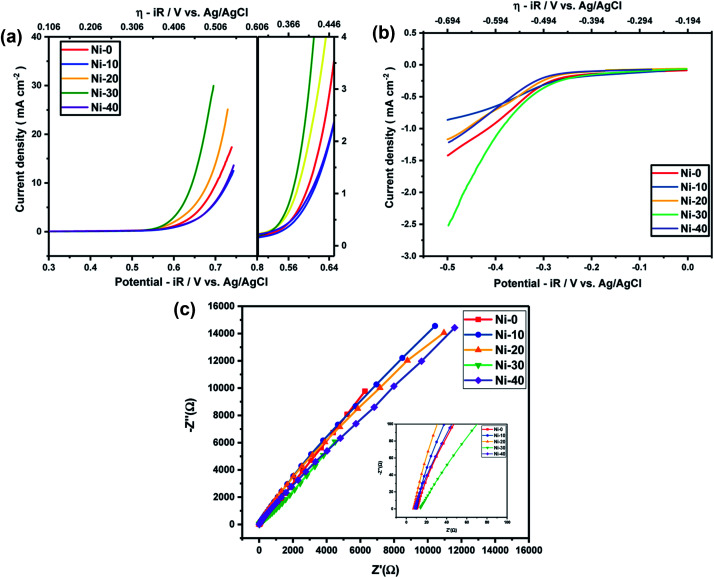
(a) OER activity of Ni_*x*_Co_1−*x*_O at 1 M KOH electrolyte. (b) ORR activity of Ni_*x*_Co_1−*x*_O at 1 M KOH electrolyte. (c) EIS measurements of Ni_*x*_Co_1−*x*_O at 1 M KOH electrolyte.

In order to know the electrochemical activities of different Ni doped cobalt oxides, linear sweep voltammetry was carried out with Ni-0 to Ni-50 samples in argon saturated 1 M KOH electrolyte and corresponding *iR* corrections were made according to EIS measurements shown in Fig. 4(c). The LS curves shown in Fig. 4(a) and (b) suggests that the highest current densities or the highest electro-catalytic activity for both OER and ORR were obtained for 30% Ni doped CoO (Ni-30) sample. The current densities for OER and ORR were on the rise with increasing doping concentration from Ni-10 to Ni-30 and drop significantly with further increase in Ni content, with Ni-40 showing even lower catalytic activities than the undoped CoO sample. However, catalytic activity of Ni-0 was better than Ni-10 in case of OER and better than Ni-10 and Ni-20 in case of ORR. This may be due to the presence of minor spinel phase in the Ni-0 and Ni-10 samples.

**Fig. 5:**
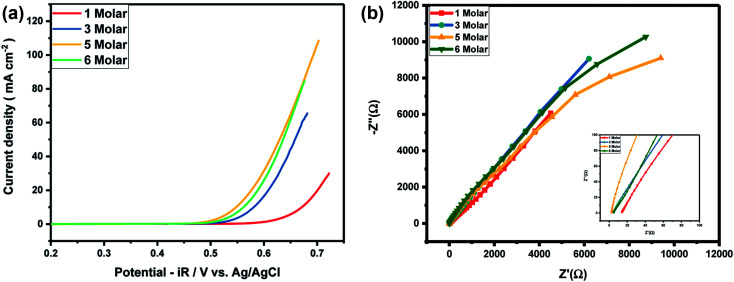
(a) Variation of OER activity of Ni_0.3_Co_0.7_O with the concentration of electrolyte. (b) EIS measurement of Ni_0.3_Co_0.7_O for different concentration of electrolyte.

**Fig. 6:**
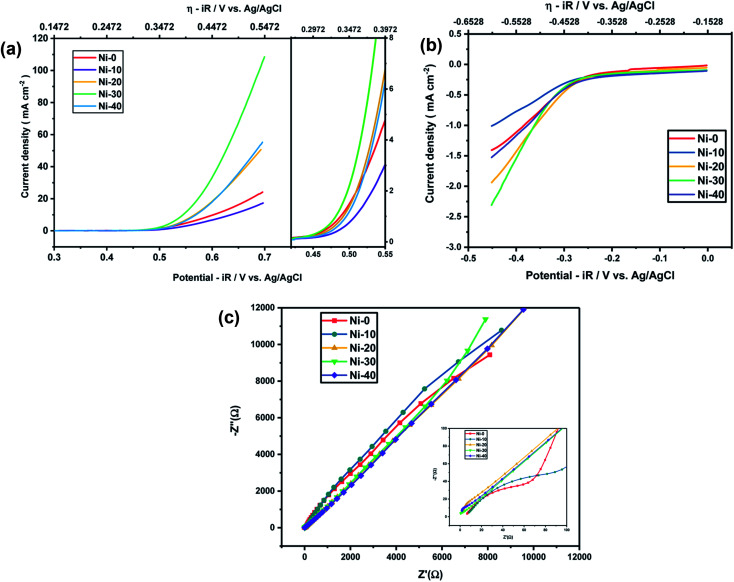
(a) OER activity of Ni_*x*_Co_1−*x*_O at 5 M KOH electrolyte. (b) ORR activity of Ni_*x*_Co_1−*x*_O at 5 M KOH electrolyte. (c) EIS measurements of Ni_*x*_Co_1−*x*_O at 5 M KOH electrolyte.

**Fig. 7:**
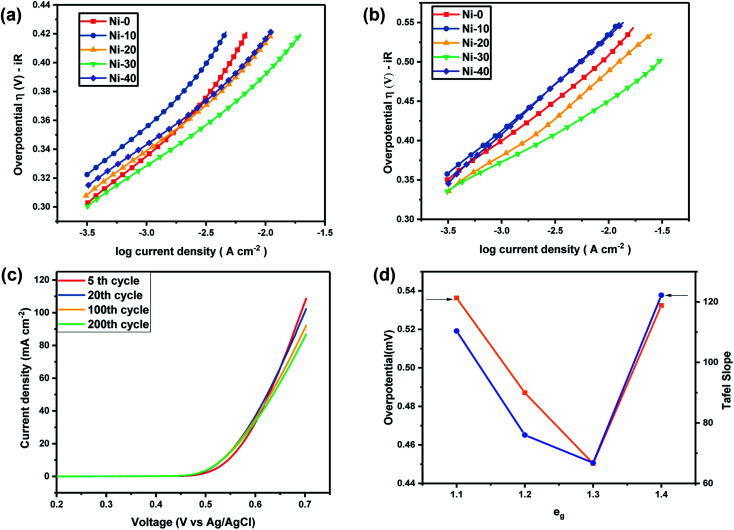
(a) Tafel plot of Ni_*x*_Co_1−*x*_O at 5 M KOH. (b) Tafel plot of Ni_*x*_Co_1−*x*_O at 1 M KOH. (c) Showing stable OER activity of Ni_0.3_Co_0.7_O up to 200 cycles. (d) Volcano graph depicting correlation of e_g_ electrons with overpotential (at 10 mA cm^−2^) and the Tafel slope at 1 M KOH electrolyte.

As Ni-30 perform superior electrochemical activity among all Ni doped CoO samples, in order to study the highest electrochemical performance of the materials, the catalytic OER activities of Ni-30 was also studied by varying the concentration of KOH electrolyte. The OER activities increases with the increasing concentration of KOH electrolyte up to 5 molar concentration, and above 5 M, the activity start degrading as shown in Fig. 5(a) and corresponding *iR* corrections were made according to EIS measurements shown in Fig. 5(b). The onset potential also decreases with the increase in concentration of KOH electrolyte upto 5 molar solutions. Therefore, electrochemical activities of all Ni doped CoO samples were studied in 5 M KOH electrolyte and the highest catalytic activity was again confirmed for Ni-30 sample for both OER and ORR as shown in Fig. 6(a) and (b). The corresponding *iR* corrections were made according to EIS measurements shown in Fig. 6(c). We have observed that in OER and ORR studies, no redox peaks were observed confirming the non capacitive behaviour and absence of high oxidation of Co and Ni in the form of M^3+^ as higher oxidation state under goes redox transformation (M^3+^/M^2+^) that generate redox peak and capacitive storage.^20^ The absence of higher oxidation state (+3/+4) of cobalt and nickel in Ni-30 sample was also confirmed by the XPS studies. To understand the electrochemical activity of the material, Tafel slope and overpotential plays an important role. Table 2 shows the Tafel slope and overpotential value corresponding to 5 M and 1 M KOH respectively. The Tafel analysis at 5 M KOH and 1 M KOH concentration for Ni-10 to Ni-40 is shown in the Fig. 7(a) and (b). Low Tafel slope is usually an indication of a good electrocatalyst and the calculated Tafel slope values may provide insightful information toward the reaction mechanism of the target system. The general OER mechanism in alkaline solution on the metal site (M) begins with a proton-coupled electron transfer from a surface-bound aqua species followed by an O–O bond formation,^21,22^ that is described as follows:

1 M–OH_2_ + OH^−^ → M–OH + H_2_O + e^−^

2 M–OH + OH^−^ → M–O* + H_2_O + e^−^

3 M–O* + OH^−^ → M–OOH + H_2_O + e^−^

4 M–OOH + OH^−^ → M–OH_2_ + H_2_O + e^−^

**Table 2:** Tafel slope and overpotential values of Ni_*x*_Co_1−*x*_O (*x* = 0 to 0.4) at 1 M and 5 M KOH

Sample	Concentration	Overpotential at 10 mA cm^−2^	Tafel slope mV dec^−1^
Ni-0	1 M KOH	0.50675	96.88
Ni-10	1 M KOH	0.5363	110.39
Ni-20	1 M KOH	0.48707	76
Ni-30	1 M KOH	0.45034	66.8
Ni-40	1 M KOH	0.53242	122.2
Ni-0	5 M KOH	0.449	70.41
Ni-10	5 M KOH	0.48291	64.84
Ni-20	5 M KOH	0.4134	53.23
Ni-30	5 M KOH	0.389	52.34
Ni-40	5 M KOH	0.4165	57.93

For the lower concentration of the electrolyte (1 M), samples Ni-0, Ni-10 and Ni-40 shows a Tafel slope close to the standard value of 96 mV dec^−1^, 110 mV dec^−1^ and 122.2 mV dec^−1^ indicating that the OER process in Ni-0, Ni-10 and Ni-40 at 1 M is limited at the first stage where the surface of catalyst was strongly bonded with –OH groups (reaction order = 1 with respect to OH^−^ species with featured Tafel slope of 120 mV dec^−1^).^23^ Tafel slope of Ni-20 and Ni-30 was found to be 76 and 66.8 mV dec^−1^ which was very close to a featured Tafel slope of 60 mV dec^−1^. Therefore for Ni-20 and Ni-30 samples, the rate determining step should be the H_2_O removal and M–O* formation.^24^ At the high concentration of electrolyte (5 M), all the samples shows a Tafel slope in the range of 57.93 to 70.41 close to a featured Tafel slope of 60 mV dec^−1^ suggesting that at higher electrolyte concentration, the rate-determining step will be H_2_O removal and M–O* formation for all the materials. Thus the OER reactions for Ni-30 in aqueous 1–5 M KOH electrolyte is controlled by the rate-determining step involving H_2_O removal and M–O* formation as given in eqn (2).^24^ This clearly suggest that with increasing OH^−^ or electrolyte concentration, electro-catalytic activity of the material will increase as demonstrated in our study. Once the entire adsorption site will be filled by OH^−^, the increase in concentration of electrolyte will have no effect on the performance of the catalyst.

The stability of the OER activity of Ni-30 catalyst is shown in Fig. 7(c). The figure shows the LS curves after (a) 5 cycles at 5 scan rates, (b) 20 cycles at 5 scan rates, (c) 100 cycles at 20 scan rate and (d) 200 cycles at 20 scan rates. In first 20 cycles, the decrease in peak current was close to 5% and further 10% of decrease was observed in 20 to 100 cycles and for 100 to 200 cycles almost 5% decrease was observed. Overall, Ni_0.3_Co_0.7_O (Ni-30) showed remarkably superior performance as an electrocatalyst for OER studies among all samples. The overpotential for Ni_0.3_Co_0.7_O (Ni-30) sample was also found ∼0.450 V for 1 M and about ∼0.389 V at 5 M concentration of the KOH electrolyte. Recently, using DFT calculation it was shown that the over-potential for OER for a given electrocatalyst cannot be below 0.37 V.^25,26^ Observed overpotential (0.389 V) of Ni-30 at 5 M electrolyte concentration is almost close to reaching the theoretical limit of 0.37 V. OER activity of different transition metal-ions containing perovskites are established in their term of e_g_ electrons present in the compound and in the volcano plot, it was established by Suntivich *et al.*, that close to 1.25 e_g_ electrons, superior OER activity and low overpotential can be achieved.^14^ The superiority Co^2+^ ions in higher OER activity were earlier confirmed by the comparative studies of ZnCo_2_O_4_, CoAl_2_O_4_ and Co_3_O_4_ in aqueous KOH electrolyte.^20^ It was shown that in rock salt structured NiCoO_2_ the Co^2+^ lies in low spin T^6^_2g_E^1^_g_ state and Ni^2+^ lies in T^6^_2g_E^2^_g_ state.^27^ Thus, the total e_g_ electrons present in the compound can be varied from e^1^_g_ to e^2^_g_ from CoO to NiO. The volcano plat representing overpotential and Tafel slope with respect to e_g_ electron present in our samples are shown in Fig. 7(d). It clearly evident from Fig. 7(d) that as the composition approaching to Ni-30 (Co_0.7_Ni_.3_O), the overall e_g_ electrons approaches to 1.3 in the compound resulting the higher OER activity.

## Conclusions

The rock-salt structure acted as a model host structure similar to perovskite where e_g_ electrons can be varied to obtain the superior electro-catalytic activity. Incorporation of nickel into CoO lattices help to stabilize the rock salt structure and tune the e_g_ electrons to develop superior OER and ORR electrocatalysts. Ni_0.3_Co_0.7_O with 1.3 e_g_ electrons showed superior electrocatalytic activity for oxygen evolution reaction. The overpotential for Ni_0.3_Co_0.7_O (Ni-30) sample was also found ∼0.450 V for 1 M and about ∼0.389 V at 5 M concentration of the KOH electrolyte. The over potential of the solid state bulk synthesised rock salt Ni_0.3_Co_0.7_O is found to be even lower than various nano-structured compounds like spinel Co_3_O_4_,^28^ and the catalytic activity is found to be even higher than that of the nano-structured Co@N–C^29^ and Ni_0.33_Co_0.67_S_2_ nanowire.^30^ The catalytic activity of rock salt Ni_0.3_Co_0.7_O is found to be better than both the rock salt parent oxide CoO (this work) and NiO.^31^ This work models a new area of tuning the e_g_ electrons in the rock salt structure similar to that perovskite structure.^14^ The nano-engineering of designated material can further improve the activity of the materials and the study will be off very much interest.

## Conflicts of interest

There is no conflicts to declare.

## Supplementary Material

RA-010-D0RA03050C-s001
