# Supplementary material for: Ni stabilized rock-salt structured CoO; Co1−xNixO: tuning of eg electrons to develop a novel OER catalyst
Source: RSC Adv. 2020 May 7;10(30):17845–53. doi: 10.1039/d0ra03050c (PMC9053581; doi:10.1039/d0ra03050c)
Supplement: RA-010-D0RA03050C-s001 [file RA-010-D0RA03050C-s001.pdf]

Electronic Supplementary Material (ESI) for RSC Advances.
This journal is © The Royal Society of Chemistry 2020

Supplementary Information:

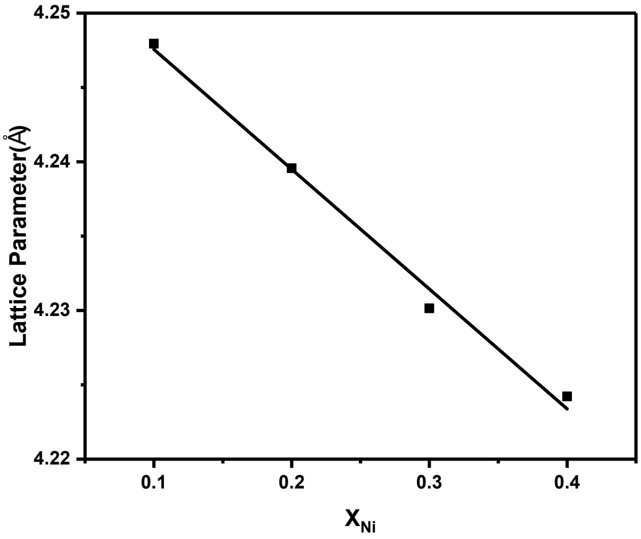

Figure S1 : Variation of the lattice parameter of $Ni_xCo_{1-x}O$ with Ni content
